# Exercise CMR: real-time assessment of cardiac performance with phase contrast imaging

**DOI:** 10.1186/1532-429X-18-S1-T2

**Published:** 2016-01-27

**Authors:** Marina Quinlan, Shareen Jaijee, Antonio de Marvao, Pawel F Tokarczuk, J Simon R Gibbs, Declan O'Regan

**Affiliations:** 1 Medical Research Council Clinical Sciences Centre, Imperial College London, London, United Kingdom; 2 National Heart and Lung Institute, Imperial College London, London, United Kingdom

## Background

Exercise CMR is an emerging technique for understanding the effects of physiological stress on the cardiovascular system in health and disease. Accurate assessment of cardiovascular performance during exercise is challenging due to high heart rates and respiratory motion therefore new imaging tools are required for real-time measurement of biventricular function. In this study we evaluated the accuracy of high-temporal resolution real-time phase contrast imaging to measure cardiac performance during progressive exercise stress.

## Methods

CMR was performed at rest and throughout exercise in 8 healthy volunteers (5 females, mean age 31 years) using a Philips 1.5T Achieva (Best, Netherlands) with a 32-channel cardiac coil. Conventional breath-hold retrospectively gated phase contrast imaging of the main pulmonary artery (MPA) and ascending thoracic aorta were acquired at rest. Exercise was performed using an MR-compatible cycle ergometer (Lode, Netherlands). Subjects were exercised in the supine position with rotational movement of pedals at a rate of 60 revolutions per minute to 40% of their maximal power output determined by prior cardiopulmonary exercise testing.

An ungated real-time echo-planar phase contrast sequence of the MPA and aorta was acquired using the following parameters: field-of-view = 300 × 300 mm; repetition time= 12 ms; echo time= 4.0 ms; flip angle = 20°; velocity encoding 200 cm/s (aorta) and 100 cm/s (MPA); temporal resolution of 50 ms.

Automated correction of voxel aliasing was performed in Matlab if required. Stroke volume index (SVI) was measured using Art Fun (Inserm, Paris) with automated in-plane vessel tracking of the MPA and aorta. Cardiac output (CO) was calculated as stroke volume × HR. Comparisons between methods were made with Bland Altman plots, differences between rest/stress conditions with the Wilcoxon signed-rank test and the agreement between MPA/Aortic flow with the correlation coefficient.

## Results

**Figure 1:**
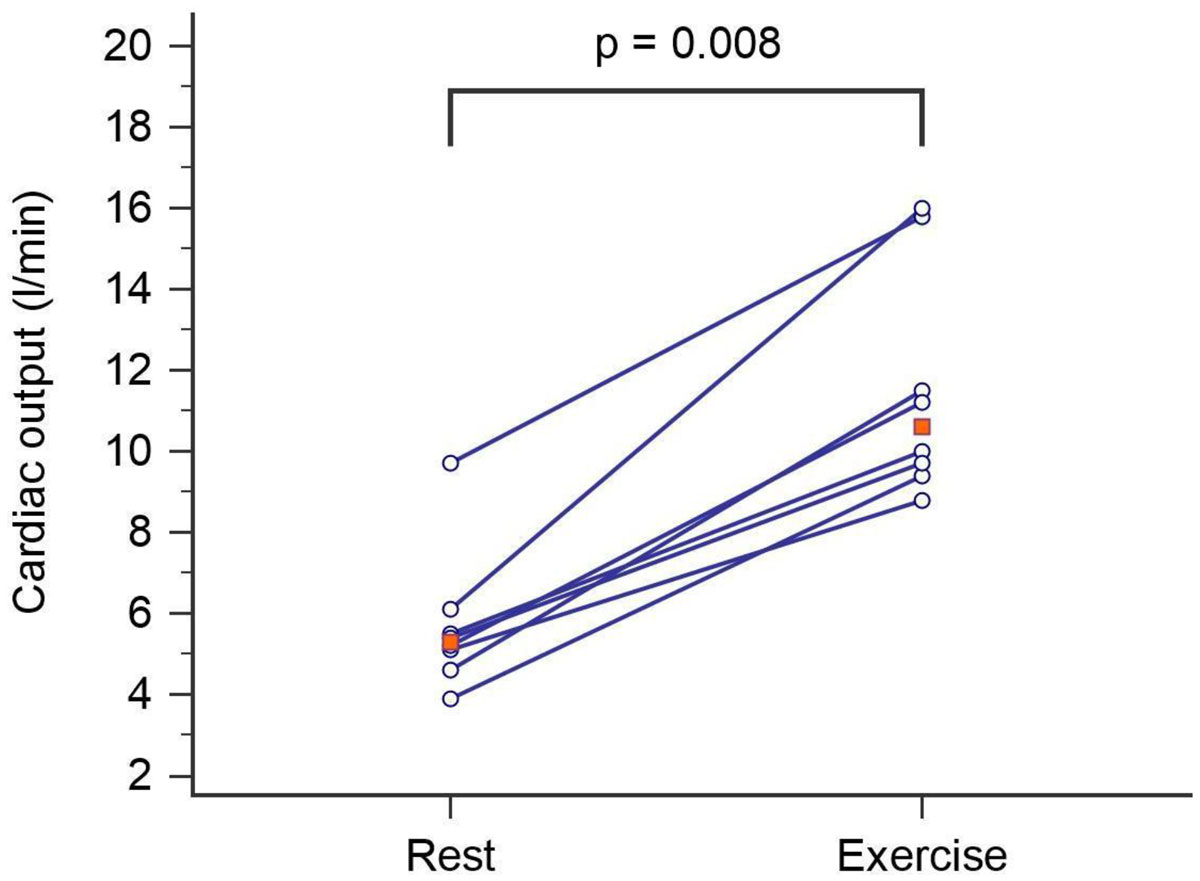
**Analysis of real-time phase contrast imaging showing flow in the aorta**. The systolic stroke volume is measured as the area under the curve (shown in blue). The change in cardiac output between rest and stress as measured with real time phase contrast imaging (red points shown median value).

All subjects tolerated exercise CMR and were able to sustain their target power output. All images were of diagnostic quality despite respiratory and thoracic motion during exercise (Figure 1). There was good agreement in SVI assessment between breath-hold and real time flow mapping at rest with no significant bias (Aorta SVI: limits of agreement -3.8 to 1.2 ml/m^2^, mean bias - 1.3 ml/m^2^; MPA SVI: limits of agreement -6.9 to 9.1 ml/m^2^, mean bias 1.1 ml/m^2^). CO measured with real-time phase contrast imaging increased between rest and stress from a median of 5.3 L/min to 10.6 L/min (p = 0.008) (Figure 2). Systemic and pulmonary SVI were highly correlated at rest (r = 0.98, p < 0.001) and during exercise (r = 0.93, p < 0.001).

## Conclusions

Real-time flow mapping offers a novel approach for measuring cardiac performance during exercise which is straightforward to acquire and analyse. Our pilot data indicates that this could be a valuable tool in the expanding role of exercise CMR.

